# London Trauma and Pre-hospital Care Conference 2011: Introduction

**DOI:** 10.1186/1757-7241-20-S1-I1

**Published:** 2012-03-22

**Authors:** David Lockey, Gareth Davies

**Affiliations:** 1 North Bristol NHS Trust & London Air Ambulance, UK; 2 Barts and the London NHS Trust and London’s Air Ambulance, UK

## 

The 5th London Trauma and Pre-hospital Care Conference took place from 22^nd^ - 24^th^ June at the Royal Geographical Society, a historic building famous for its role in the golden age of British exploration. Exactly 100 years ago the Royal Geographical Society launched Captain Scott’s doomed expedition to the South Pole, to which he was beaten by the great Norwegian explorer Roald Amundsen. This was perhaps a fitting venue to welcome a large delegation from the Norwegian Air Ambulance who have contributed much to the academic and social content of the conference. Over 400 participants from 14 countries attended the conference to explore current and evolving concepts in trauma and pre-hospital care.

The large Ondaatje Lecture theatre hosted the main trauma conference on the first two days of the conference. The third day concentrated on pre-hospital care and major incidents. The format was of short lectures with three specific questions to be answered by each speaker. In addition there was one eponymous lecture, two keynote addresses and a debate. In addition to the main lecture theatre a parallel session was held each day. These included Core Trauma Topics, Trauma Systems Forum and a Trauma Research day. A study day on trauma and pre-hospital care for medical students was held around the venue on the first conference day and scientific abstracts were presented orally on the second evening.

The next London Trauma and Pre-hospital Care Conference will be held at the same venue from 4^th^ to 7^th^ December 2012. The event will for the first time include a whole day on air ambulance work hosted by the Norwegian Air Ambulance.

